# The complexity, challenges and benefits of comparing two transporter classification systems in TCDB and Pfam

**DOI:** 10.1093/bib/bbu053

**Published:** 2015-01-21

**Authors:** Zachary Chiang, Ake Vastermark, Marco Punta, Penelope C. Coggill, Jaina Mistry, Robert D. Finn, Milton H. Saier

**Keywords:** TCDB, Pfam, transport protein classification, data integration

## Abstract

Transport systems comprise roughly 10% of all proteins in a cell, playing critical roles in many processes. Improving and expanding their classification is an important goal that can affect studies ranging from comparative genomics to potential drug target searches. It is not surprising that different classification systems for transport proteins have arisen, be it within a specialized database, focused on this functional class of proteins, or as part of a broader classification system for all proteins. Two such databases are the Transporter Classification Database (TCDB) and the Protein family (Pfam) database. As part of a long-term endeavor to improve consistency between the two classification systems, we have compared transporter annotations in the two databases to understand the rationale for differences and to improve both systems. Differences sometimes reflect the fact that one database has a particular transporter family while the other does not. Differing family definitions and hierarchical organizations were reconciled, resulting in recognition of 69 Pfam ‘Domains of Unknown Function’, which proved to be transport protein families to be renamed using TCDB annotations. Of over 400 potential new Pfam families identified from TCDB, 10% have already been added to Pfam, and TCDB has created 60 new entries based on Pfam data. This work, for the first time, reveals the benefits of comprehensive database comparisons and explains the differences between Pfam and TCDB.

## Introduction

Protein classification databases organize entries into coherent schemas that typically highlight evolutionary or functional relationships between different protein entries. The information stored in these databases is widely used for predicting function of experimentally uncharacterized proteins and for automatic annotation of whole proteomes by transferring functional annotations as a result of homology [1]. Numerous databases exist that focus on different but overlapping themes (e.g. sequence similarity, structural similarity, molecular function, evolutionary pathway) or have different resolution (e.g. protein domains and functional subfamilies versus large multi-component families) [2–10].

In this contribution, we describe some examples of differences in how proteins and protein families have been classified within the Protein family (Pfam) database (pfam.xfam.org) [11] and the Transporter Classification Database (TCDB) (www.tcdb.org) [12–14]. We present examples of how Pfam and TCDB differ and how some of the differences can be explained. The process of resolving differences is time-consuming, and some are impossible to resolve owing to philosophical differences between the two databases. Both databases rely on manual curation for functional annotation, but while Pfam aims to comprehensively classify all protein or protein domain homologous relationships, TCDB focuses on the functional/phylogenetic classification of whole transport systems. Pfam contains 14 488 literature references over the whole protein family spectrum, while TCDB includes 9814 literature references for transport systems alone.

The fundamental difference between the two resources is that Pfam groups protein regions (or domains) into homologous families and homologous families into clans, while TCDB classifies transport systems initially based on function and mechanism with homology playing a secondary role. The basic units of Transporter Classification (TC) are complete transport systems that often include nonhomologous domains and/or proteins. These units are then organized into a five-level hierarchy (class, subclass, family, subfamily and transport system, with a superfamily hyperlink that is independent of the hierarchical system). These conceptual differences make mapping between Pfam and TCDB complex with many-to-many relationships between entries.

## Results

Our first goal towards understanding the differences between Pfam and TCDB was to identify entries in one classification system that were missing in the other. Thus, we started by asking the simple question: how many of the transport proteins classified by TCDB were not found in a Pfam family?

### Transporter families absent in Pfam

TCDB proteins lacking a match to Pfam (version 27.0) would constitute prime candidates for generation of new Pfam transport-related families. Of 11 382 protein sequences in TCDB, 1122 proteins were not annotated by Pfam (see Methods). To minimize the complexity of the many-to-one relationships posed by this difference between TCDB and Pfam, we considered only one representative protein for each group that shared the first four levels of TC classification. We filtered out all proteins belonging to class 9, which contains established and putative transport systems lacking a known mechanism of action. Note that for simplicity, we ignored the fact that nonhomologous subunits of multi-component systems can be found in the same TCDB (sub)family (see Discussion). This may lead to underrepresentation of the total number of potential new families. An iterative application of this approach, after the addition of new families, could eventually lead to all potential new families being identified. After the addition of the new families to Pfam and regeneration of a new profile hidden Markov model (HMM) library, the pfam_scan searches can be repeated to identify any TCDB proteins still not found in Pfam. In other words, if a protein with TC numbers 1.A.2.1.X (where X stands for any TC transport system) has been ignored in the first iteration because of the presence of a nonhomologous subunit with the same first four TC numbers, it will be picked up by the second iteration. Thus, pfam_scan will find any nonmatching protein with TC number 1.A.2.1.X, and this can be repeated until no additional proteins can be found.

This procedure resulted in a final set of 407 protein sequences, after a single round of the analysis method described above, which could be used to initiate a new protein family in Pfam. To check the potential of these protein sequences being built into Pfam families, we focused on the proteins that produced at least 100 hits in our reference database after two iterations of jackhmmer (see Methods), that is, the ones most likely to produce the largest families. These amounted to 196 proteins overall; see Supplementary Materials File TCDB-for-Pfam.xlsx). To date, Pfam curators have processed 53 entries from this list. Of these, 40 new families have been built. Of the remaining possibilities, 13 sequences did not lead to the construction of new Pfam families for a variety of reasons including (1) they matched Pfam families that were independently built after release 27.0 but before this analysis, (2) they matched one of the families just built from the TCDB list (i.e. they were homologous to some other protein in the list even though they had a different level 4 classification in the TC system) (3) they corresponded to obsolete UniProtKB sequences or (4) they turned out to be outliers of existing families. Examples of new Pfam families created from TCDB include, among others, the Fungal Potassium Channel (F-Kch) Family (TC# 1.A.88; preliminary Pfam accession ahead of Pfam release 28.0: PF16944) and the Chloroplast Envelope Anion Channel-forming (Tic110) Family (TC# 1.A.18; 3.A.9; preliminary Pfam accession ahead of Pfam release 28.0: PF16940).

### Using TCDB annotations to improve Pfam

Approximately one-third of the protein families in Pfam lack any associated functional or experimental characterization. Many of these families fall into two groups that are referred to as Domains of Unknown Function (DUFs) and Uncharacterized Protein Families (UPFs, derived from Swiss-Prot). Often, experimental data are published subsequent to the generation of a Pfam entry. Unfortunately, Pfam lacks a sophisticated mechanism necessary for routinely matching published experimental data to database entries and relies on biocurators to annotate the entries; this requires significant effort and resources. Consequently, some DUFs and UPFs may remain functionally unannotated after the publication of relevant experimental data. An adjunct to identifying TCDB proteins that lack a Pfam match is the identification of DUFs and UPFs that match sequences where TCDB has functional data.

Within the set of Pfam families that matched proteins in the TCDB sequence set (classes 1–5 only), there were 126 DUFs and UPFs (see Supplementary Materials File DUF-UPF-list.txt). However, the mere presence of a Pfam entry matching a protein classified by TCDB is insufficient for the transfer of annotation. For example, a DUF may represent a soluble domain of unknown function in an otherwise characterized transporter protein. In other cases, there may not be enough experimental evidence in TCDB to warrant renaming a DUF. So far, curators have analyzed 92 of these Pfam entries, finding that 69 could be renamed and annotated according to information found in TCDB.

An alternative approach could be to use UniProt/Swiss-Prot directly for adding functional information to DUFs. The problem with UniProt/Swiss-Prot to Pfam data transfer is that UniProt curation does not always reflect the latest functional data, and it annotates proteins rather than domains, making automatic transfer impossible. Families that match newly annotated proteins in UniProt/Swiss-Prot could, of course, be flagged and checked systematically for new domain annotation, but that would still require a considerable amount of work on the part of Pfam curators. Given the limited resources, the Pfam solution for keeping annotations up to date in the past few years has been instead to crowd-source annotation via Wikipedia. The better linking between Pfam and TCDB should alert UniProt/Swiss-Prot curators to the specialist TCDB resource and accelerate discovery of the relevant literature.

The next goal was to identify differences in annotations and assignments between Pfam and TCDB and then to determine reasons underlying those differences. We decided to take a top-down approach to the comparison, starting with the broadest classification-level that could be compared, the Pfam clan and TCDB superfamily levels. Comparisons were then conducted secondarily at the family level.

### Resolving inconsistencies between Pfam clans and TCDB superfamilies

Challenges in this comparison involved the fact that TC families include multi-component systems with multi-domain protein members that are nonhomologous. In addition, Pfam clans may include functionally diverse, albeit homologous, families, which in TCDB would be classified in systems with different TC numbers (see Discussion).

In Table S1, we list five Pfam families identified by the comparative analysis (described in Methods) as putative new members of existing Pfam clans. These Pfam families were cross-examined with the Pfam entries that belonged to the original Pfam clan, corresponding to the equivalent TCDB superfamily, to ensure that they were homologous. Other families that were suggested for inclusion into transporter protein clans turned out to be either soluble domains or N-/C-terminal extensions of families already in the clan. In these cases, no further action was taken. One family, Connexin_CCC (PF10582), was eliminated and its N-terminal companion, Connexin (PF00876), was extended to cover the entire transmembrane domain. Finally, three families [Innexin (PF00876), Connexin (PF00029) and DUF3733 (PF12534)] that had been suggested by comparative analysis to be part of the Leucine Rich Repeat (CL0022) clan were instead added to the Transporter (CL0375) clan based on Pfam criteria for clan membership.

Similarly, we identified TC superfamilies that could be expanded based on information from Pfam clan annotations, once the necessary statistical criteria had been satisfied (Table S2). TCDB’s statistical criteria for homology detection currently require the alignments to have an alignment score of greater than 14 standard deviations (SD) compared with the average alignment score in a score distribution obtained from GSAT [15] after randomly shuffling the sequences at least 1000 times. Also, the alignment must be of sufficient length (60 amino acids) and contain at least two aligned transmembrane segments (TMSs) that have equivalent positions in the predicted protein topology [12–14] [Methods section ‘c’, Transport Pfam-1 (RepFam-1)]. The TC Amino acid/Polyamine/organoCation (APC) superfamily [16], as an example, matches Pfam CL0062 (similarly named APC), but seven families of CL0062 did not match family members in the APC superfamily in TCDB (2.A.120, 2.A.26, 2.A.55, 2.A.114, 2.A.72, 2.A.46 and 2.A.31), leading to expansion of the TC APC superfamily [17].

From the Pfam clans so far investigated, 60 new sequences were added to TCDB. However, the complexity of the annotation comparison will require months of work to complete, and this task must be ongoing, as Pfam and TCDB are continually being updated. Within TCDB, there are several new superfamilies that have not yet been investigated using the time-consuming statistical procedures used by the TC group, and they therefore have yet to be added to TCDB.

Indeed, when using Pfam as a guide, it is important that TCDB independently satisfy their own homology criteria. In the future, we expect the number of new incorporations from such a comparison to be significantly smaller so that the TCDB curation process remains a viable and scalable methodology.

### Cross-validating family annotations in Pfam and TCDB

As seen in the previous section for clans and superfamilies, the family-level annotations in Pfam and TCDB are not always easily compared. Similarly, many of the observed inconsistencies can be ascribed to the different approaches to protein classification adopted by the two databases. Nevertheless, some differences can help highlight areas in which annotation could be improved. It has become clear that Pfam families are often less functionally specific than their TC counterparts. In Table S3, for example, we report on a number of manually identified cases taken from the Major Facilitator Superfamily, Mitochondrial Carrier (MC), Major Intrinsic Protein, P-type ATPase, APC and DMT superfamilies, where the TC system appears to have higher functional specificity than Pfam. This is to be expected, partly because the sensitivity of Hidden Markov ModelER 3 profile HMMs makes it difficult to separate closely related functional subfamilies, and the aim of Pfam is to try to make the families as broad as possible, i.e. model all or most homologous sequences within a single entry.

### Re-annotating DMT Superfamily constituents

In this section, we describe comparisons between the TCDB Drug/Metabolite Transporter (DMT) superfamily (TC# 2.A.7) and the Pfam DMT clan (CL0184). The comparisons demonstrate the benefits of working together to improve annotation of the DMT superfamily/clan [18].

Naming of the Pfam DMT clan (CL0184) was originally guided by TCDB [18]. Many of the entries were pre-existing families within Pfam, originating from different sources. Since then, the DMT clan in Pfam and the DMT superfamily in TCDB have been maintained and added to independently, leading to the current situation (as of Pfam 27.0) where there is ‘core’ agreement between the DMT clan and superfamily, but also several differences. Consequently, the DMT proteins constitute an interesting case. Note that in the case of the DMT superfamily, both the TCDB superfamily and family levels correspond to a single TC number (2.A.7), i.e. they have been collapsed into a single level, while the 4th level of the TCDB annotation is in this case used to indicate subfamilies. Also, families in the Pfam DMT clan are meant to exclusively represent the transmembrane domain of the transport proteins, thus excluding any additional soluble domain that may occur.

At the DMT clan/superfamily level, we observed good correspondence between Pfam and TCDB, but there were a few exceptions. Members of the TCDB Ca^2+^ Homeostasis Protein family (Csg2; TC# 2.A.7.27) were not present in Pfam (e.g. UniProtKB accession P35206); hence, a new family was created (CSG2 or Ceramide synthase regulator, provisional accession ahead of Pfam release 28.0: PF16965) and added to the Pfam DMT clan. Conversely, five Pfam families in the DMT clan [Cation_efflux (PF01545), CRCB (PF02537), DUF486 (PF04342), UPF0546 (PF10639) and Zip (PF02535)] contained proteins not found in TCDB. The Cation_efflux family (PF01545) has recently been removed from the Pfam DMT clan because of lack of homology evidence. Two of the above families (DUF486 and UPF0546) were added to the DMT superfamily (2.A.7.34 and 2.A.7.32, respectively) while the other three were added to TCDB but not to the DMT superfamily, due to insufficient statistical evidence of homology. Indeed, with the current TCDB cutoff for homology of 14 S.D. (see above), there is insufficient evidence that CrcB (1.A.43) should be linked to the DMT superfamily in TCDB, although evidence was borderline for Zip (comparison score of 13.7 S.D.). Interestingly, according to TCDB criteria, there is also insufficient evidence that Cation_efflux (comparison score of 12.4 S.D.) should be included in the DMT superfamily. TCDB may create a super-superfamily hyperlink to DMT for Zip if more evidence becomes available.

**Figure 1:**
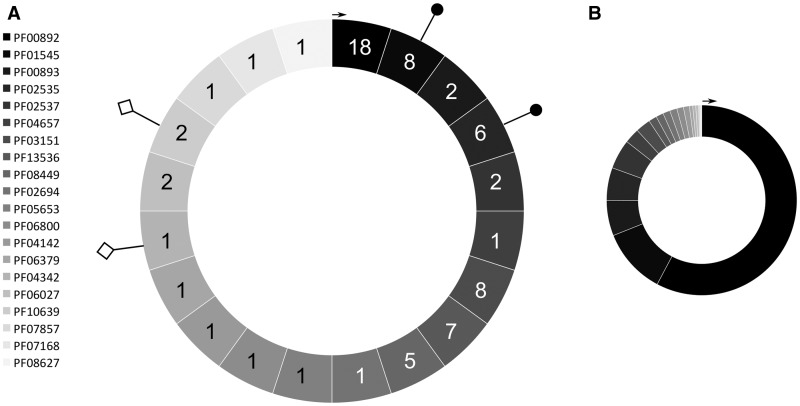
Graphical overview of the mapping of the Pfam DMT clan to TCDB’s DMT superfamily. (**A**) Each segment in the circle represents one of the 20 Pfam families found in the DMT clan (release 27.0). To the left of the circle is the full list of accession numbers for families in this clan. The first segment at 12 o’clock (with black background) corresponds to the first family in the list starting from the top (i.e. PF00892). PF00892 maps to 18 subfamilies in the DMT superfamily in TCDB, hence the number 18 reported in this segment. Moving clockwise from PF00892, we find all other families in the list, from PF01545 (mapping to eight subfamilies in TCDB) to PF08627 (mapping to a single TCDB subfamily). Different shades of gray are meant to help identify the families. The diamond-shaped lollipops indicate Pfam families that have been used to create new subfamilies in the TCDB DMT superfamily: PF04342 was used to create subfamily 2.A.7.34 and PF10639 to create subfamilies 2.A.7.32 and 2.A.7.33. The pin-shaped lollipops represent families for which TCDB is considering creating hyperlinks to the DMT superfamily (Zip (PF02535) and Cation efflux (PF01545), see main text). (**B**) Each segment in the circle represents one of the 20 Pfam families found in the DMT clan. The order of the families is the same as outlined in (A); however, segments have sizes that correspond to the percentage of DMT domains found in that family (of the total number of domains for the whole DMT clan). For example, family PF00892 comprises about 66 000 domains, or almost 58% of the clan’s domains.

Below the clan/superfamily level, the relationships between TCDB (sub)families and Pfam families are complex as represented in Figure 1A. Each segment of the chart represents a Pfam family in the DMT clan as of Pfam release 27.0 (20 families overall), and the integer displayed in the segment in the schematic figure corresponds to the number of TCDB subfamilies that map to the Pfam entry (TCDB DMT superfamily as of May 2014, 34 subfamilies). Pfam family PF00892, for example, maps to 18 different TCDB subfamilies. Featured subfamilies include, among others, 2.A.7.2–4, 7, 16–18, 20, 22–24 and 28–31, which are diverse in their sequences and functions. The functional diversity of this Pfam family can be better understood by looking at the pie chart in Figure 1B, which shows how this family covers close to 58% of the domains in the entire Pfam clan. Other families cover between ∼0.02% (PF08627) and ∼11% (PF01545) of the clan. This is a typical situation for Pfam clans that represent large superfamilies characterized by a continuum of similar sequences.

If we add up the number of TCDB subfamilies that map to the 20 Pfam families in Figure 1A, the total sum is not 34 but 70, showing that TCDB subfamilies can map to more than one Pfam family. Many of these one-to-many relationships between TCDB subfamilies and Pfam families involve family EamA (PF00892). In this context, it should be remembered that domain sequences in Pfam clans may have significant sequence similarity to more than one family in the clan, but they are assigned to the most significant single family (the one with the smallest E-value), a process termed ‘clan competing’.

Understanding the underlying reasons for the differences between Pfam and TCDB is nontrivial, as the reasons are unique to each superfamily/clan. One of the difficulties with the DMT clan concerns the different topologies of the member proteins. DMT proteins are known to have 2, 4 (2 + 2), 5 and 10 (5 + 5) TMS topologies [19], where 2 + 2 and 5 + 5 indicate a tandem repeat of 2 and 5 TMS units, respectively [18, 20]. Some Pfam family models that map to the 5 TMS tandem repeat type cover both repeat units [e.g. the UAA (PF08449), RhaT (PF06379) and Ureide_permease (PF07168) families], while others, such as the EamA (PF00892) and EmrE families (PF13536) [21] cover 4 TMSs in a single copy of the repeat. In some proteins, single repeat models combine to produce mixed annotation (e.g. one N-terminal EmrE domain and one C-terminal EamA domain). This is, in part, a consequence of the internal competition between models in the same clan.

It was clear from examining transmembrane predictions that many Pfam families were insufficiently long to match all relevant TMSs (Table S4). Consequently, several Pfam families have been extended to cover the entirety of the transmembrane region. However, despite the best efforts of Pfam curators, using the local–local matching strategy between the sequence and profile HMM, it was not always possible to correctly match the different topologies. For example, in the case of EmrE, a single match covers TMSs 1-4, but there can be multiple matches between the sequence and the profile HMM. Splitting tandem repeat families into their individual repeated domains is often not performed because splitting repeat units usually does not return all sequences retrieved by the longer full tandem repeat models, thus lowering coverage. Finally, because of the close similarities between some of the families in the DMT clan, splitting according to single repeat units tends to erroneously generate more hybrid annotations such as the ones observed for the EamA and EmrE families.

Review of the DMT clan in Pfam led to further changes besides the ones described above: a subfamily of plant Triose Phosphate Transporters (PF03151) was created as PUNUT (PF16913); five DUF/UPF families in the clan (function unknown) were renamed: DUF606 (now called DMT_YdcZ, PF04657), DUF486 (DMT_6, PF04342), DUF914 (SLC35F, PF06027), DUF1632 (TMEM144, PF07857) and UPF0546 (TMEM234, PF10639); functional annotations were added to several Pfam families in the clan, thanks to literature-based experimental evidence present in the TCDB DMT superfamily.

## Discussion

Pfam and TCDB feature a number of important differences in terms of both goals and methodology, which had to be taken into consideration when comparing the two resources. One of the most important distinctions between the two databases concerns the scopes of their classifications. Pfam is a general-purpose database whose main goal is to be comprehensive, that is, to classify a large portion of the protein space. To this end, Pfam families are usually designed to be as far-reaching as possible. Homology is no guarantee of functional similarity [1]; consequently Pfam families can be functionally diverse. In addition to families, Pfam defines clans, which constitute the upper tier of a two-level hierarchy. Families are grouped into a clan when there is sufficient evidence that they are homologous. Evidence can come from a combination of sequence, structural and functional information. While some families within the same clan represent distinct functional subgroups, many families are added to clans to increase sequence coverage when previous models were not sensitive enough to detect all homologues. Finally, experimental functional knowledge of one or more members is not a prerequisite for a family being part of Pfam. Indeed, close to 3800 families in Pfam 27.0 are currently named DUFs [22–24] or UPFs, although for some of these, functional information has become available after the family’s generation.

**Figure 2:**
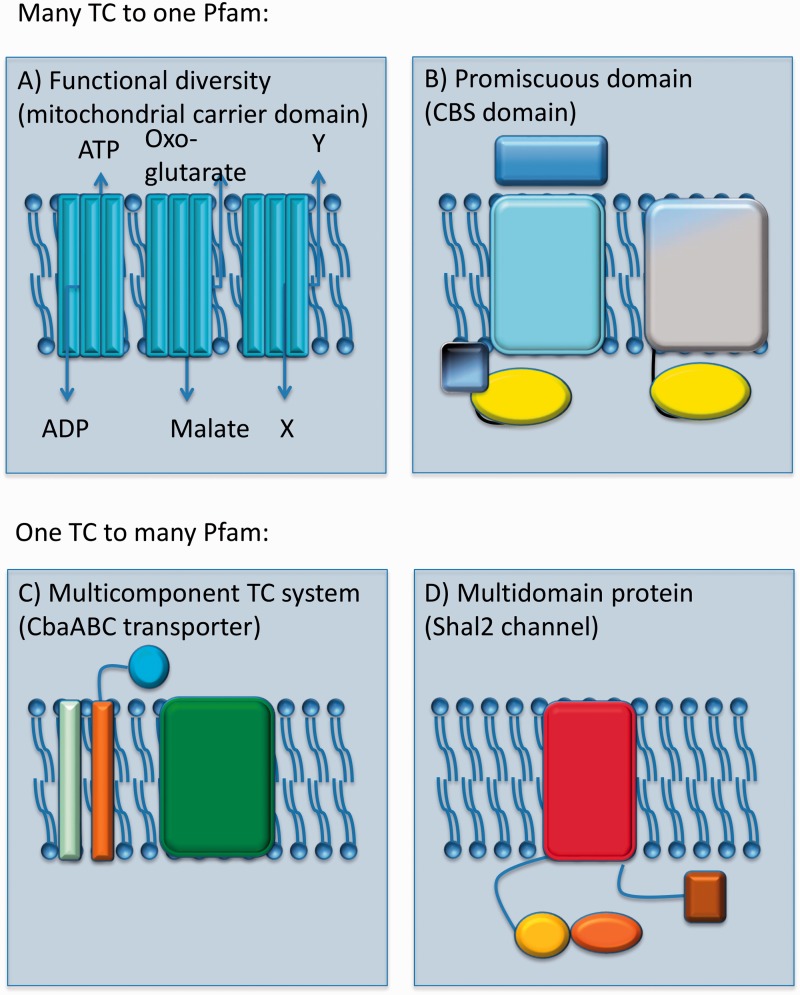
Complex relationships between families in TCDB and Pfam. (**A**) The same domain in Pfam, the 2 TMS repeat unit Mito_carr (PF00153), covers sequences that are parts of functionally diverse subfamilies in TCDB: 2.A.29.1, three copies of Mito_carr; 2.A.29.2, also three copies of Mito_carr, etc. (**B**) The same domain in Pfam, CBS (PF00571) (soluble), is found in systems belonging to two different TCDB classes (primary active and secondary carriers): 3.A.1.12.1 {on the left: three components, [OpuAC (PF04069)—soluble], [ABC_tran (PF00005)—soluble and CBS (PF00571)—soluble], [BPD_transp_1 (PF00528)—membrane inserted]} and 2.A.49.1.1 {on the right: one component, [Voltage_CLC (PF00654)—multispanning membrane-inserted] and [CBS (PF00571)—soluble]}. (**C**) The Cytochrome Ba3 oxidase three component system is represented by a single entry in TCDB (3.D.4.2.1), while in Pfam each of its three constituent chains maps to one or more families annotated as evolutionary unrelated. From left to right: *CoxIIa* (PF08113), COX2-transmemb (PF09125) and COX2 (PF00116, soluble), COX1 (PF00115). (**D**) A single component system in TCDB (VIC superfamily member, 1.A.1.2.3) maps to multiple domains in Pfam: Shal-type (PF11601, N-terminal), BTB_2 (PF02214), Ion_trans (PF00520, membrane-inserted) and DUF3399 (PF11879, C-terminal); only Ion_trans is a transmembrane domain.

TCDB is a database of transport systems based on a five-level hierarchical classification. The hierarchy is not symmetrical because, depending on the transport system being considered, the same level can represent a superfamily or a family. In contrast to Pfam, which defines homology-based families that can be functionally diverse, the main goal of TCDB is to provide a precise functional/phylogenetic separation between its families and subfamilies. This is the origin of numerous one-to-many relationships between Pfam families and TCDB subfamilies and families, as in the case of the DMT clan/superfamily discussed above. In another example, a single Pfam family [the MC family (PF00153)] corresponds to 30 functionally distinct subfamilies in TCDB (TC# 2.A.29.1–30) [25, 26] (Figure 2A).

It is important to note that in TCDB, for the purpose of classification, the function of multi-component systems requires inclusion of nonhomologous proteins and protein domains under a single TC number. For example, the Proton-translocating Cytochrome Oxidase (COX) Superfamily (TC# 3.D.4) includes under a single TC number all COX subunits (e.g. the complete transport system of 3.D.4.1.1 consisting of SoxABC), three proteins that are not homologous to each other [27, 28]. In contrast, Pfam classifies them into different unrelated families (Figure 2C). In other cases, TCDB may place homologous proteins into different classes, where ‘class’ is the first, more generic tier in the classification system. For example, homologues of Tic110 are found in families that are part of both the primary active transporter class, e.g. the Chloroplast Envelope Protein Translocase (CEPT or Tic-Toc) Family (TC# 3.A.9), and the Channels/Pores class, e.g. the Chloroplast Envelope Anion Channel-forming Tic110 (Tic110) Family (TC# 1.A.18) [29]. A single Pfam family, however, covers proteins from both the CEPT and the Tic110 TC families (preliminary Pfam accession ahead of Pfam release 28.0: PF16940).

As noted above, TCDB classifies full-length proteins, whereas Pfam targets conserved protein regions irrespective of protein length. This reflects another fundamental difference in the databases’ goals: while TCDB aims to transfer the full functional apparatus of a protein to all other family members, Pfam establishes relationships between individual functional modules that may be found in proteins with different functions. These differences are additional sources of one-to-many relationships between TCDB and Pfam. Still another example, the potassium voltage-gated channel protein Shal (UniProtKB P17971, TC# 1.A.1.2.3), part of the TCDB VIC superfamily [30, 31], maps to four families in Pfam: Shal-type (PF11601), BTB/POZ (PF02214), Ion_trans (PF00520) and DUF3399 (PF11879) (Figure 2D). In a contrasting example, homologous Cystathionine Beta Synthase (CBS) domains, all mapping to Pfam family CBS (PF00571), are present in proteins of TCDB transporter families 2.A.49, 3.A.1.12 and 9.B.149 (Figure 2B).

The examples presented in this contribution illustrate the complexity of comparing two resources such as TCDB and Pfam. Both teams believe that the comparisons have revealed where both classification systems can be improved. Furthermore, ensuring greater consistency facilitates better cross-linking between Pfam and TCDB and makes the transitions from one to the other less confusing for users. Using the software tools developed, we will continue to compare and refine the two systems of transport protein classification to ensure continued data exchange and to synergize our respective biocuration efforts. We hope that this study will serve as an example for other databases, leading to tighter integration of similar-theme resources and better validation of their respective contents.

## Methods

### Identifying TC proteins/families that are not yet classified by Pfam and Pfam DUF/UPF families with available annotation in TCDB

We used a May 2014 TCDB version containing 11 382 protein sequences and Pfam release 27.0 containing 14 831 families. The pfam_scan program (available from ftp://ftp.ebi.ac.uk/pub/databases/Pfam/Tools/), a wrapper around the HMMER software (HMMER version 3.1b1, http://hmmer.janelia.org), was used to find all TCDB protein matches (TC classes 1–5 and 8) to families in Pfam release 27.0. The pfam_scan program was used in default mode (–cut_ga option that uses the family-specific Pfam gathering thresholds to establish significance). Sequences lacking a match to Pfam were then used as a starting sequence for an iterative jackhmmer search (run with default options) against pfamseq27, which is a sequence database derived from UniProtKB version 2012_06 [32]. Note that, to reduce redundancy, we considered only one representative protein for each group that shared the first four levels of TC classification. The 196 sequences that produced over 100 matches after two jackhmmer iterations were then considered for inclusion into Pfam. Pfam_scan further allowed us to identify 126 DUFs/UPFs families in Pfam that matched TC proteins in classes 1–5.

### SubClass-Viewer-1 and RepFam-1

For the purpose of family comparison, the TCDB team has developed two scripts, SubClass-Viewer-1 (SCV1) and RepPfam-1 (available on request), which can help to automatically identify cases similar to those found in Table S3 across the whole spectrum of Pfam and TC families. SCV1 allows plotting TC families within a selected subclass (the second level of the TCDB hierarchical system) against the Pfam families that match them, thus helping to uncover one-to-many relationships between the families in the two databases.

SCV1 draws a two-dimensional array, plotting TC families within a selected subclass on the y-axis (left), and Pfam families that have matches to them on the x-axis (on top of the graphical output; by default using E-value threshold of 1e-5). For each cell where there is a match, a color scale is applied where a shade of red is assigned on a 10-increment scale from light to dark. Each nuance represents the average fraction of sequences within the TC family that have a Pfam match. Note that TC families with more than one average hit per sequence (i.e. average fraction >1) are assigned to the 0.9–1.0 color scale bin. The program, which outputs Support Vector Graphics (SVG), has been designed to identify families that have unique matches between the TC and Pfam systems. It also identifies cases where Pfam families break up between TC families (i.e. where the TC system has higher resolution), and can be used to recommend splitting and joining of families of both systems. For this purpose, a separate script, SubClass-Viewer-SVG-reader, was developed that can load an SVG-formatted file, generated by SCV1, and analyze it for patterns, such as cells that are ‘isolated’ (the cell does not have any horizontal or vertical neighbors) in both the x- and the y-dimensions of the plot. A nice feature is that the script that analyzes the SVG file and reports families and matches that fulfill different criteria uses coordinates that directly correspond to positions in the SVG. They are reported when the graphics file is opened in a program (such as Inkscape), facilitating direct interpretation of the graphical output. An advantage of this dual interface approach is the opportunity for the user to edit the graphics file before analysis, manually filtering out hits that may be irrelevant before automatic processing of the file.

The RepPfam-1 script allows the viewing of how multiple Pfam hits to a given TC family are distributed in relation to the location of the predicted transmembrane helices (using TransMembrane Hidden Markov Model (TMHMM) version 2 [33]; http://www.cbs.dtu.dk/services/TMHMM/). It provides estimates of average numbers of TMSs within a certain group of Pfam hits in both tabular and graphical form. This can be a useful tool in refining transmembrane domain boundaries of Pfam families as shown in the main text for the DMT clan.

### Clan-Viewer-1

The 702 Pfam families that match to the 798 families in classes 1–5 of TCDB are represented in Clan-Viewer-1 (available on request) as a 27 by 27 matrix, that is, the square matrix of lowest order that can contain 702 elements. Elements in the matrix can be highlighted to manually examine the overlap between a given TC superfamily and different Pfam clans. For example, each of the 77 Pfam clans that map to TCDB proteins can be highlighted relative to the 47 superfamilies they correspond to. The user can choose a superfamily, and then scroll through the graphical output of the program to identify which clan(s) match(es) the superfamily. A version of this program (index-03) producing tabular output was used to generate the results in Table S1. First, index-03 runs all TCDB sequences against Pfam HMMs (using hmmscan, E-value significance cutoff of 1e-25). For each superfamily in TCDB the script identifies all matching Pfam families along with the clans they belong to. In building Table S1, we considered only TC superfamilies where at least 60% of matching Pfam families were part of the same clan. For these cases, index-03 can be used to propose changes to the clan classification of the families not found in the most represented clan. As an example, the Sodium:Neurotransmitter symporter Family Pfam family (not in a clan in release 27.0) was proposed to join the APC clan as it mapped to six sequences from TC 2.A.22, which is a member of the APC superfamily in TCDB. Index-03 does not explicitly handle multi-component systems, but the user can choose to supply a version of TCDB that has been pre-filtered against such systems, or (as was the case in Table S1) manually remove potential false positives.

### Automatic identification of proteins to be entered into TCDB

We ran all TCDB sequences against Pfam version 27.0 HMMs (pfam_scan with default parameters). For each TC superfamily, we selected all Pfam clans for which more than half of the clan’s family members hit the TC superfamily. For each of these clans, we identified all Pfam families that did not hit any TC sequence. For each of these Pfam families, we downloaded sequence representatives from the Pfam Web site. We then ran TC BLAST on each sequence to count how many TMSs each one contained and to find the sequences’ closest matches in TCDB. We considered for addition to TCDB only cases where the Pfam sequence contained the same (or similar; ±1) number of TMSs as its closest match in TCDB and for which the E-value for the alignment between the Pfam sequence and the TCDB closest match was in the range 0.1–1 × 10^−^^7^ (Table S2). The reason why there is a lower limit on the E-value is because we are particularly interested in the distant matches, which represent novel subfamilies, not currently in TCDB. The scripts used in this pipeline are included in the supplementary materials.

**Key Points**

- We have (1) initiated a comparative analysis of transport proteins in the TCDB and Pfam databases and (2) developed approaches for data exchange in these two databases. Thanks to the ongoing collaboration between the TCDB and Pfam groups, our analyses have led to beneficial changes to both resources. We hope that this study will benefit other database developers that face similar integration problems with overlapping resources.

## Supplementary data

Supplementary data are available online at http://bib.oxfordjournals.org/.

## Funding

This work was supported by NIH Grant GM077402 (to M.H.S.) and funding from the Wellcome Trust [WT077044/Z/05/Z to P.C.C., and M.P.], the European Molecular Biology Laboratory, European Bioinformatics Institute (EMBL-EBI) (to J.M. and R.D.F).

## Supplementary Material

Supplementary Data
