# Supplementary material for: The complexity, challenges and benefits of comparing two transporter classification systems in TCDB and Pfam
Source: Brief Bioinform. 2015 Jan 21;16(5):865–72. doi: 10.1093/bib/bbu053 (PMC4570203; doi:10.1093/bib/bbu053)
Supplement: Supplementary Data [file supp_16_5_865__index.html]

The complexity, challenges and benefits of comparing two transporter classification systems in TCDB and Pfam — The complexity, challenges and benefits of comparing two transporter classification systems in TCDB and Pfam — Supplementary Data 

# The complexity, challenges and benefits of comparing two transporter classification systems in TCDB and Pfam

## Supplementary Data

files

**Files in this Data Supplement:**

- Supplementary Data - pdf file
- Supplementary Data - txt file
- Supplementary Data - docx file
- Supplementary Data - docx file
- Supplementary Data - docx file
- Supplementary Data - docx file
- Supplementary Data - xlsx file
