# Supplementary material for: The complexity, challenges and benefits of comparing two transporter classification systems in TCDB and Pfam
Source: Brief Bioinform. 2015 Jan 21;16(5):865–72. doi: 10.1093/bib/bbu053 (PMC4570203; doi:10.1093/bib/bbu053)
Supplement: Supplementary Data [file supp_bbu053_compare_pfam_tcdb.pdf]

```
#compare_pfam_tcdb.pl

#Usage: perl compare_pfam_tcdb.pl > sample_clan_list.txt
#Input: Expects a list of pfam clans as input.  Each clan should #be
on a new line and be in the format CL0000.
#Output: Produces a file called final_table.txt

#Implementation

#The script's implementation is broken into three separate parts:

#Part 1 - Reads in the input file and creates a list of families in
each pfam clan provided.
#Part 2 - Downloads a list of proteins in each family and uses TC
BLAST to find each one's closest match in TCDB.
#Part 3 - Filters each clan's TC BLAST results and uses Protein BLAST
to remove similar proteins.

#Filtering Criteria

# - Selects TC BLAST results with an e-value between 0.01 and e^(-8)
# - Uses HMMTOP to select proteins with an equal or greater number of
TMSs than its closest match
# - Removes any proteins containing a trailing "ase" to ensure no
enzymes appear

#Dependencies

#Perl Modules - LWP::Simple, WWW::Mechanize
#Other - BLAST

#Written by Zachary Chiang
#Last Updated: 12/2/14

# !/usr/bin/perl

use strict;
use constant { TRUE => 1, FALSE => 0 };
use WWW::Mechanize;
use LWP::Simple;

# new mechanize instance
my $mech = WWW::Mechanize->new;

# part 1 - for each clan provided, create a list of families it
contains

# clan to family relationships file
open (CLANS, 'Pfam-A.clans.tsv');

# part 1 output file
open (CLANS_AND_FAMS, ">clans_and_fams.txt");
```

```
# stores clans
my @pfam_clans = ();

# iterate through STDIN, should have a clan on each line
while(<STDIN>){

     # stores current clan
    my $clan = "";

     # add clan to array if not already there
    if ($_ =~ /(CL[0-9]{4})/p){
        $clan = $1;
        my $in_array = FALSE;
        foreach my $pfam_clan (@pfam_clans){
            if ($clan eq $pfam_clan){
                     $in_array = TRUE;
                 }
        }
        if ($in_array eq FALSE){
                 push (@pfam_clans, $clan);
             }
    }
}

# iterate through each clan
foreach my $pfam_clan (sort @pfam_clans){

     # seek to beginning of clan to family relationship file
    seek CLANS, 0, 0;

     # initialize array to store families and set count to 0
    my $num_fam = 0;
    my @pfam_families = ();

     # iterate through relationship file
    while(<CLANS>){

          # add families in clan to array and increment count
        if ($_ =~ /$pfam_clan/p && $_ =~ /(PF[0-9]{5})/p){
            push (@pfam_families, $1);
            $num_fam++;
        }
    }

     # print clan and all families to output file
    if ($num_fam > 0) {
        print CLANS_AND_FAMS $pfam_clan . " " . $num_fam . "\n";
        foreach my $pfam_family (sort @pfam_families){
            print CLANS_AND_FAMS "\t" . $pfam_family . "\n";
        }
```

```
    }
}

# close part 1 files
close CLANS;
close CLANS_AND_FAMS;

# part 2 - blast each family's sequences against TCDB

# part 1 output file
open (CLANS_AND_FAMS, 'clans_and_fams.txt');

# part 2 output file containing full table of blast results
#open (FULL_TABLE, '>full_table.txt');

# initilize part 2 variables
my $clan = "";
my @families = ();
my $count = 0;
#my $status = FALSE;

# iterate through part 1 output
while (<CLANS_AND_FAMS>){

      # new clan, get sequences for previous clan
      if ($_ =~ /(CL[0-9]{4})/){

            &get_proteins;

            # reset families and clan
            @families = ();
            $clan = $1;

      # family of current clan, push to array
      } elsif ($_ =~ /(PF[0-9]{5})/){
            push (@families, $1);
      }
}

# get sequences for last clan
&get_proteins;

close CLANS_AND_FAMS;
#close FULL_TABLE;

# part 3 - combine into final table and blast proteins
open (FINAL, '>final_table.txt');

# iterate through each .txt file in data folder
my $dir = './data';
foreach my $file (glob("$dir/*.txt")){
```

```
# store current clan
my $clan = "";

# open current table
open (FILE1, $file);

# stores proteins
my @proteins = ();

# iterate through table
while (<FILE1>){

      # found clan, open file to store results
      if ($_ =~ /(CL[0-9]{4})/){
            open (FILE2, "+>./results/".$1."_result.txt");
            $clan = $_;
            print FILE2 $_;
            print $_;

      # line is part of table
      } else {

            # split line into array by tabs
            my @line = split(/\t/);

            # initialize table values
            my $evalue = 0;
            my $num_tms = 0;
            my $hit_num_tms = 0;
            my $accession_num = "";
            my $annotation = TRUE;

            # find and store values
            if ($line[2] =~ /([0-9A-Z]{6})/){
                  $accession_num = $1;
            }

            if ($line[3] =~ /([0-9]+) TMSs/){
                  $num_tms = $1;
            }

            if ($line[5] =~ /([0-9]+) TMSs/){
                  $hit_num_tms = $1;
            }

            if ($line[6] =~ /e(-[0-9]+)/){
                  $evalue = exp($1);
            } else {
                  $evalue = $line[6];
            }

            if ($line[7] =~ /ase/){
```

```
                    $annotation = FALSE;
                }

                # use criteria to filter lines
                if ($num_tms >= $hit_num_tms && $num_tms > 1 &&
$hit_num_tms > 1 && $evalue < 0.01 && $evalue > exp(-8) && $annotation
eq TRUE){
                    print FILE2 $_;
                    push (@proteins, $accession_num);
                }
            }
        }

        # marker and count for blasting proteins
        my $current_protein = 0;
        my $count = 1;

        # iterate until all proteins have been blasted
        while ($current_protein < scalar @proteins){

            # reset count
            $count = 1;

            # open file for subject and get current protein sequence
            open (SUBJECT, ">subject.txt");
            my $url_1 =
'http://www.uniprot.org/uniprot/'.@proteins[$current_protein].'.fasta'
;
            $mech->get($url_1);

            # print sequence to file
            for ($_ = $mech->content){
                if ($_ =~ s/([A-Z])\n/$1/g){
                    print SUBJECT $_;
                }
            }

            # iterate through query proteins
            while ($current_protein + $count < scalar @proteins){

                # open file for query and get next protein sequence
                open (QUERY, ">query.txt");
                my $url_2 =
'http://www.uniprot.org/uniprot/'.@proteins[$current_protein +
$count].'.fasta';
                $mech->get($url_2);

                # print sequence to file
                for ($_ = $mech->content){
                    if ($_ =~ s/([A-Z])\n/$1/g){
                        print QUERY $_;
                    }
```

```
                }

                # debug statement
                print @proteins[$current_protein] . " - " .
@proteins[$current_protein + $count];

                # run blastp with subject and query
                system ("blastp -query query.txt -subject subject.txt
-out blast_results.txt");

                # open results file
                my $evalue = 0;
                open (RESULTS, "blast_results.txt");

                # get evalue
                while(<RESULTS>){
                     if ($_ =~ /Expect = (.*),/){
                          $evalue = $1;
                          print ": " . $1;
                          last;
                     }
                }

                # filter by evalue
                if ($evalue =~ /e-([0-9]+)/ && $1 > 8){
                     splice(@proteins, $current_protein + $count, 1);
                     print " DELETED";
                } else {
                     $count++;
                }

                # debug message
                print "\n";

                # close query file
                close QUERY;

           }

           # increment and close subject file
           $current_protein++;
           close SUBJECT;
      }

      # print clan
      if (scalar @proteins > 0){
           print FINAL $clan;
      }

      # print all proteins to final table
      foreach my $protein (@proteins){
           seek FILE2, 0, 0;
```

```
            while (<FILE2>){
                  if ($_ =~ /$protein/){
                        print FINAL $_;
                        last;
                  }
            }
      }

}


sub get_proteins {

      # check for at least one family
      if (scalar @families ne 0){

            # open file
            open (TABLE, '>./data/' . $clan . '_output.txt');

            # print clan to table
            print TABLE $clan . "\n";

            # iterate through families
            foreach my $family (sort @families){

                  # get sequences from family and initialize temp file
                  my $url =
'http://pfam.sanger.ac.uk/family/'.$family.'/alignment/seed/format?for
mat=fasta&alnType=seed&order=t&case=l&gaps=default&download=0';
                  my $file = 'temp.txt';

                  # get url contents and open temp
                  my $status = getstore($url, $file) or die 'Unable to
get page';
                  open (TEMP, 'temp.txt');

                  # store uniprot numbers
                  my @uniprot_nums = ();

                  # interate through temp file and grab new uniprot
numbers
                  while (<TEMP>){
                        if ($_ =~ />([A-Z 0-9]{6})_.*\/(.*)/){
                              my $in_array = FALSE;

                              foreach my $uniprot_num (@uniprot_nums){
                                    if ($uniprot_num eq $1){
                                          $in_array = TRUE;
                                          last;
                                    }
                              }
                              if ($in_array eq FALSE){
```

```
                              push (@uniprot_nums, $1);
                         }
                    }
               }

               # close temp file
               close TEMP;

               # tc blast each unique uniprot number
               foreach my $uniprot_num (sort @uniprot_nums){
                    &tc_blast($uniprot_num, $family);
               }
          }
          # close table
          close TABLE;
     }
}

sub tc_blast {

     # initialize variables
     my $uniprot_num = $_[0];
     my $family = $_[1];
     my $tms_num = 0;
     my $hit_tms_num = 0;
     my $nearest_hit = "No TCDB hits";
     my $e_value = "N/A";
     my $annotation = "";

     # submit form with uniprot number as field
     $mech->get('http://tcdb.org/progs/blast.php');
     $mech->submit_form(
          with_fields => {
               "SEQUENCE" => $uniprot_num,
          }
     );

     # open second temp file and print contents
     open (TEMP2, '+>temp2.txt');
     print TEMP2 $mech->content(format => 'text') . "\n";
     seek TEMP2, 0, 0;

     # iterate through temp file and grab values
     while (<TEMP2>){
          if ($_ =~ /\(HMMTOP\): ([0-9]+)/){
               $tms_num = $1;
               if ($_ =~ /Query: .+_[0-9A-Z]+ (.+) OS/){
                    $annotation = $1;
               }
          } elsif ($_ =~ /([0-9]+ TMSs)\t([0-9]+\.[A-
Z]\..+)\t.+\t.+\t(.+)$/){
```

```
                if ($_ =~ /([0-9]+ TMSs)\t([0-9]+\.[A-
Z]\..+)\t.+\t.+\t(.+)$/){
                        $hit_tms_num = $1;
                        $nearest_hit = $2;
                        $e_value = $3;

                        # print values to table
                        print TABLE "\t" . $family . "\t" . $uniprot_num
. "\t" . $tms_num . " TMSs \t"
                        . $nearest_hit . "\t" . $hit_tms_num . "\t" .
$e_value . "\t" . $annotation . "\n";
                        last;
                }
            }
      }

      # close temp file
      close TEMP2;
}
```
