# Supplementary material for: The complexity, challenges and benefits of comparing two transporter classification systems in TCDB and Pfam
Source: Brief Bioinform. 2015 Jan 21;16(5):865–72. doi: 10.1093/bib/bbu053 (PMC4570203; doi:10.1093/bib/bbu053)
Supplement: Supplementary Data [file supp_bbu053_Table_S1.docx]

**Table S1.** *Pfam families that have been added to clans based on comparative analysis between Pfam and TCDB.*

| **Pfam family, clanless** | **Proposed clan to join** | **Proposed reason for joining** | **Action taken by Pfam** |
| --- | --- | --- | --- |
| SNF (PF00209) | APC (CL0062) | Hits 2.A.22.1-6 | Added to clan CL0062 |
| BCCT (PF02028) | APC (CL0062) | Hits 2.A.15.1-2 | Added to clan CL0062 |
| EXS (PF03124) | IT (CL0182) | Hits 2.A.94.1 | Added to clan CL0182 |
| ANKH (PF07260) | MviN_MATE (CL0222) | Hits 2.A.66.9 of the MOP superfamily | Added to clan CL0222 |
| Aegerolysin (PF06355) | CDC (CL0293) | Hits 1.C.97.4,6 of the MACPF superfamily | Added to clan CL0293 |
