# Supplementary material for: The complexity, challenges and benefits of comparing two transporter classification systems in TCDB and Pfam
Source: Brief Bioinform. 2015 Jan 21;16(5):865–72. doi: 10.1093/bib/bbu053 (PMC4570203; doi:10.1093/bib/bbu053)
Supplement: Supplementary Data [file supp_bbu053_Table_S2.docx]

**Table S2.** *Proteins entered into TCDB from Pfam using the automatic identification protocol described in Methods (section d).*

| **Pfam family** | **Pfam protein** | **Topology** | **Annotation** | **Entered into TCDB** |
| --- | --- | --- | --- | --- |
| PF12040 | Q7NL23 | 6 TMSs | Gll1303 protein | 3.A.1.132.10 |
| PF12040 | Q21EL5 | 6 TMSs | ABC-2 type transport system permease protein | 3.A.1.132.11 |
| PF04093 | Q9K8H7 | 5 TMSs | Cell-shape determining protein | 9.B.157.1.2 |
| PF07399 | Q9F206 | 12 TMSs | NhaE, new subfamily, remotely related to 2.A.111.1 | 2.A.111.2.1 |
| PF07456 | A5N7N1 | 5 TMSs | Uncharacterized, new subfamily, remotely related to 2.A.87.1.1 | 2.A.87.3.1 |
| PF02674 | A8ESJ6 | 4 TMSs | New family was created (CvpA). | 9.B.160.1.1 |
| PF07456 | A7VSQ7 | 5 TMSs | Entered into the previously created new subfamily. | 2.A.87.3.2 |
| PF07974 | Q80YS4 | 2 TMSs | Tek from mouse. | 1.A.87.2.4 |
| PF07974 | Q86AS3 | 2 TMSs | High cysteine content. Note that in this case two proteins from the same Pfam family have been entered into different TC classes | 9.B.87.1.2 |
| PF00008 | Q0NGD8 | 2 TMSs | Secreted epidermal growth-factor-like protein | 9.B.87.1.4 |
| PF12661 | Q4RSA9 | 2 TMSs | Uncharacterized protein | 9.B.87.1.5 |
| PF12662 | B8LPX7 | 2 TMSs | Putative uncharacterized protein | 9.B.87.1.6 |
| PF12662 | Q339K4 | 2 TMSs | Putative vacuolar sorting receptor protein homolog | 9.B.87.1.7 |
| PF07686 | Q8UWK1 | 2 TMSs | Novel immune type receptor 4 | 8.A.17.3.1 |
| PF07090 | A1ACJ3 | 3 TMSs | Uncharacterized protein | 1.A.13.5.1 |
| PF03239 | Q9Y8Z4 | 6 TMSs | Ftr1 protein | 2.A.108.2.8 |
| PF06779 | Q9I3T2 | 12 TMSs | Uncharacterized MFS permease | 2.A.1.51.4 |
| PF06813 | Q8H132 | 12 TMSs | UMF23 permease | 2.A.1.75.5 |
| PF07690 | P95852 | 12 TMSs | MFS porter | 2.A.1.46.8 |
| PF07690 | Q8TZJ0 | 12 TMSs | MFS porter | 2.A.1.2.88 |
| PF07690 | Q97XW7 | 12 TMSs | MFS porter | 2.A.1.1.118 |
| PF07690 | Q986D7 | 12 TMSs | MFS porter | 2.A.1.8.16 |
| PF07690 | Q986N1 | 12 TMSs | MFS porter | 2.A.1.36.5 |
| PF07690 | Q9I0P8 | 12 TMSs | Uncharacterized protein | 2.A.1.11.8 |
| PF07690 | Q9KYI2 | 12 TMSs | MFS porter | 2.A.1.2.89 |
| PF07690 | Q9L8Q4 | 12 TMSs | MFS porter | 2.A.1.13.21 |
| PF07690 | Q9RSM6 | 14 TMSs | MFS porter | 2.A.1.3.67 |
| PF11700 | Q0BZH6 | 12 TMSs | MFS porter | 2.A.1.24.6 |
| PF00515 | O26730 | 9 TMSs | Uncharacterized protein | 9.B.2.1.4 |
| PF07721 | Q887X9 | 2 TMSs | Uncharacterized protein | 9.B.159.6.7 |
| PF13181 | A5CZ17 | 16 TMSs | Uncharacterized protein | 9.B.67.1.3 |
| PF13414 | A0YQV7 | 11 TMSs | Uncharacterized protein | 9.B.142.4.4 |
| PF13431 | Q1INJ4 | 10 TMSs | TRP repeat containing protein | 9.B.147.1.8 |
| PF14559 | A6DTT7 | 13 TMSs | Uncharacterized protein | 9.B.67.1.4 |
| PF00560 | O33932 | 2 TMSs | InlD protein | 8.A.43.1.3 |
| PF00560 | Q8WRE3 | 2 TMSs | Toll9 | 8.A.43.1.4 |
| PF00560 | Q92EV6 | 2 TMSs | Uncharacterized protein | 8.A.43.1.5 |
| PF00560 | Q9FZB9 | 2 TMSs | Uncharacterized protein with an ATP binding domain | 1.A.87.2.5 |
| PF00560 | Q9VGH2 | 2 TMSs | Uncharacterized protein | 8.A.43.1.6 |
| PF12799 | B9GAY9 | 6 TMSs | Uncharacterized ADP-binding protein. May be involved in defense responses | 1.A.25.3.4 |
| PF12799 | C3XYZ1 | 2 TMSs | Uncharacterized protein | 8.A.43.1.7 |
| PF13401 | A3YI93 | 2 TMSs | Uncharacterized protein | 9.B.42.1.2 |
| PF13476 | A5KMI4 | 2 TMSs | Uncharacterized protein | 1.C.105.2.8 |
| PF02518 | O86861 | 6 TMSs | AgrC | 9.B.14.1.3 |
| PF07885 | Q8U058 | 3 TMSs | Uncharacterized protein | 1.A.1.29.2 |
| PF07885 | Q92ZS8 | 4 TMSs | Uncharacterized protein | 1.A.1.27.3 |
| PF13520 | C3N8F9 | 14 TMSs | Putative amino acid porter | 2.A.3.8.30 |
| PF13520 | D0NVC6 | 12 TMSs | APC family member | 2.A.3.3.20 |
| PF13520 | Q6F2A1 | 12 TMSs | Putative polyamine transporter | 2.A.3.8.31 |
| PF03547 | Q98L58 | 10 TMSs | Putative MdcF malonate transporter | 2.A.69.3.6 |
| PF00711 | Q9DG57 | 2 TMSs | β-defensin prepropeptide | 1.C.85.3.2 |
| A0LKG2 | A0LKG2 | 3 TMSs | Outer membrane factor. May function with an ABC exporter (A0LKG3/A0LKG4) and a membrane fusion protein (A0LKG1) (based on genomic context). | 1.B.17.4.1 |
| A3XMH6 | A3XMH6 | 4 TMSs | Uncharacterized protein | 9.B.176.1.1 |
| PF13632 | A7EIH8 | 8 TMSs | Putative glycosyl transferase | 4.D.3.2.1 |
| PF13632 | A7BVJ0 | 4 TMSs | Uncharacterized protein | 4.D.1.1.6 |
| PF13641 | O67594 | 6 TMSs | Alginate synthesis-related protein | 4.D.1.1.7 |
