# Supplementary material for: The complexity, challenges and benefits of comparing two transporter classification systems in TCDB and Pfam
Source: Brief Bioinform. 2015 Jan 21;16(5):865–72. doi: 10.1093/bib/bbu053 (PMC4570203; doi:10.1093/bib/bbu053)
Supplement: Supplementary Data [file supp_bbu053_Table_S3.docx]

**Table S3.** *Examples of large Pfam families featuring one-to-many relationships to TCDB families.*

| **Pfam family** | **TC category of cluster** | **Resolution problem** |
| --- | --- | --- |
| Sugar_tr (PF00083) | MFS (2.A.1) | Does not distinguish between ~7 related MFS families in TCDB. |
| MFS_1 (PF07690) | MFS (2.A.1) | Does not distinguish between ~35 related MFS families in TCDB. |
| Mito_carr (PF00153) | MC (2.A.29) | Does not distinguish between ~28 closely related MC families in TCDB. |
| MIP (PF00230) | MIP  (1.A.8) | Does not distinguish between ~17 closely related MIP families. |
| Hydrolase (PF00702), E1-E2_ATPase (PF00122) (multi-domain scenario) | P-ATPase (3.A.3) | Does not distinguish between ~14 closely related P-ATPase families. |
| Aa_trans (PF01490) | AAAP (2.A.18) | Does not distinguish between ~9 AAAP families. |
| AA_permease (PF00324), AA_permease_2 (PF13520) | APC (2.A.3), CCC (2.A.30) | While 5 TC subfamilies preferentially match PF13520, and 6 subfamilies preferentially match PF00324, 7 subfamilies have competing preferences for both. |
