# Supplementary material for: The complexity, challenges and benefits of comparing two transporter classification systems in TCDB and Pfam
Source: Brief Bioinform. 2015 Jan 21;16(5):865–72. doi: 10.1093/bib/bbu053 (PMC4570203; doi:10.1093/bib/bbu053)
Supplement: Supplementary Data [file supp_bbu053_Table_S4.docx]

**Table S4.** *Changes to boundaries of families in the Pfam DMT clan to better reflect known domain boundaries in TCDB.*

| **Pfam family** | **TCDB equivalences** | **Topology** | **Problem identified** | **Pfam actions** |
| --- | --- | --- | --- | --- |
| EamA (PF00892) | DMT (2.A.7.2-4, 7, 16-18, 20, 22-24, 28-31) | 5 TMSs (e.g. 2.A.7.2), 5+5 TMSs (e.g. 2.A.7.3-4) | Hits TMSs 2-5 (2.A.7.2); Hits only TMSs 7-10 in the second domain (2.A.7.3); Hits TMSs 2-5 and 7-10 (2.A.7.4) | Extended to include all 5 TMSs in the single repeat unit. |
| Nuc_sug_transp (PF04142) | DMT (2.A.7.12) | 5+5 TMs | Hits TMSs 3-10 as one unit; misses the inherent repeat domain units. | Extended to include all 10 TMSs. |
| TPT (PF03151) | DMT (2.A.7.9,14-16) | 5+5 TMSs | Hits TMSs 6-10 only. | Extended to cover all TM helices. Created an additional family to increase coverage (PF16913). |
| Ureide_permease (PF07168) | DMT (2.A.7.19) | 5+5 TMSs | Hits TMSs 1-8 as one unit. | Extended model to include all 10 TMSs. |
| DUF914 (PF06027) | DMT (2.A.7.24.9-12) | 5+5 TMSs | Hits 1-10 as one unit. | Moved family ID from DUF914 to SLC35F. Retained as 10 TMSs. |
| DUF1632 (PF07857) | DMT (2.A.7.8) | 5+5 TMSs | Hits 1-7 as one unit. | Extended to include all 10 TMSs, changed name to TMEM144. |
| EmrE (PF13536) | DMT (2.A.7.7) | 5+5 TMSs | Hits as 5+5, EmrE+EamA | Rebuilt. Now covers full tandem repeat. |
| CRT-like (PF08627) | DMT (2.A.7.20.2) | 7+3 TMSs | Hits TMSs 1-2 as CRT-like, and 8-10 as a TPT domain | Extended to include all TMSs. |
